# Effect of Heat‐Processed Corn and a Carbohydrase Enzyme in Mash Diets on Nutrient Digestibility, Growth Performance and Ileal Microbial Count in Broiler Chicks During Finisher Period

**DOI:** 10.1002/vms3.70320

**Published:** 2025-05-19

**Authors:** Mohsen Teymouri, Hosna Hajati, Ahmad Hassanabadi, Abolghasem Golian

**Affiliations:** ^1^ Department of Animal Science, Faculty of Agriculture Ferdowsi University of Mashhad Mashhad Iran; ^2^ Agricultural Research, Education, and Extension Organization (AREEO) Animal Science Research Institute of Iran Karaj Iran

**Keywords:** apparent total tract retention | broiler | enzyme | processing temperature

## Abstract

**Background:**

Feed processing improves nutrient digestibility by reducing anti‐nutritional factors and enhancing starch availability.

**Objectives:**

This study evaluated the effects of heat‐processed corn and enzyme supplementation in mash diets on nutrient digestibility, growth performance, intestinal morphology and ileal microbial count of broilers.

**Methods:**

Two trials were conducted. In Trial 1, the apparent metabolisable energy (AME) and apparent total tract retention (ATTR) of corn's nitrogen (N), dry matter (DM), calcium (Ca) and total phosphorus (TP) were determined. In Trial 2, a completely randomised design with a 4 × 2 factorial arrangement was used to assess the effects of processing temperatures (unprocessed or processed at 55°C, 70°C and 85°C) and enzyme supplementation (0 or 0.5 g/kg diet). A total of 480 25‐day‐old Ross 308 broilers were assigned to 48 experimental units with 8 treatments, 6 replicates and 10 chicks each.

**Results:**

Heat processing at 85°C significantly increased the AME value of corn compared to unprocessed corn or corn processed at 55°C (*p* < 0.05). Additionally, processing at 55°C improved Ca digestibility compared to unprocessed corn (*p* < 0.05). Neither processing nor enzyme supplementation significantly affected the AMEn value or ATTR of crude protein (CP), DM or TP during Days 26‒28. Broilers fed diets with corn conditioned at 55°C or 70°C, both groups without enzyme supplementation, showed the lowest feed conversion ratio (FCR) (*p* < 0.05). Villi height was greater in broilers receiving processed corn compared to those consuming unprocessed corn (*p* < 0.05). Conditioning corn at 55°C resulted in a lower ileal *Clostridium* count at 42 d.

**Conclusion:**

Conditioning of corn at 55°C without enzyme supplementation in mash diets improved Ca utilisation, FCR and jejunal villus height while decreasing the ileal *Clostridium* count in broiler chicks.

## Graphical abstract

Corn processed at 55°C when used in mash form finishing broilers diet, improved Ca utilisation and FCR and decreased the *Clostridium* count in ileal contents. The addition of exogenous enzyme to the mash diet containing conditioned corn at 70°C led to an improvement in FCR. The processing of corn improved the height of intestinal villi.

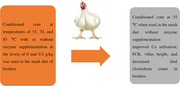

## 1 Introduction

Intestinal health critically influences broiler performance, and the period from hatching to slaughter is essential to consider. To achieve a healthy gut, poultry nutritionists strive to formulate diets that optimise the structure and function of the digestive tract, its microflora, and the associated immune system during different periods of broiler life. Different feed processing methods, such as pelleting and heat conditioning, as well as feed additives like exogenous enzymes, can enhance gut health and nutrient efficiency in poultry.

Nowadays, corn is the main feed ingredient for supplying energy and CP for broilers. Starch and non‐starch polysaccharides (NSP) are two substrates in cereals, such as corn, and nutritionists pay much attention to their amount and solubility. Starch is the largest energy‐producing component in broilers diets. Thus, maximising starch digestion is crucial for achieving optimal energy efficiency. Various feed processing methods have been shown to improve corn starch utilisation (Teymouri and Hassanabadi 2021).

Mechanical, thermal and moisture treatments of corn have been previously reported (Hoffman et al. 2012; Castrillo et al. 2013; Attar et al. 2017; Karami et al. 2018). Thermal conditioning effectively eliminates harmful substances like trypsin inhibitors (Avilés‐Gaxiola et al. 2018), makes feed more hygienic (Boroojeni et al. 2014b) and increases starch digestion (Svihus 2014; Zaefarian et al. 2015).

In the poultry feed processing industry, there are two types of conditioners: conventional conditioners and super‐conditioners. Conventional conditioning is applied in the pellet production line, where ground feed ingredients are heated at 75°C−85°C for 15–20 s (Lewis et al. 2015). According to Attar et al. (2017), a formulated diet is entered into a separate super‐conditioner designed with a diagonal angle to increase the filling and retention time. This method has advantages, such as increasing the amount of gelatinised starch, which enhances digestibility. Nutrient digestibility is greater for super‐conditioned ingredients (Attar et al. 2017). On the other hand, the main purpose of adding enzymes to the diet is to decrease the anti‐nutritional content of ingredients and improve the nutrient utilisation efficiency. High dietary NSP content can increase gut viscosity, negatively impacting nutrient digestibility and increasing nutrient encapsulation, which reduces nutrient availability. Dietary NSP‐degrading enzymes reduce the viscosity of the digesta in the small intestine, improving digesta passage and nutrient digestion rates, while also limiting substrate availability for fermenting microorganisms. Therefore, adding exogenous enzymes to decrease digesta viscosity can help control the growth of anaerobic bacteria such as *Clostridium perfringens* or *Escherichia coli* (Melo Duran 2021). The use of exogenous enzymes, such as xylanase, not only reduces environmental pollution by decreasing nutrient excretion through excreta but also positively influences intestinal health and growth performance (Melo Duran 2021). Xylanase degrades endosperm cell walls, releasing encapsulated nutrients, and has a prebiotic effect due to the production of short‐chain oligosaccharides from the polysaccharide arabinoxylan (Khadem et al. 2016). Moreover, arabinoxylo‐oligosaccharides can be effective prebiotics in poultry diets. The degradation of dietary arabinoxylan may release substrates that promote beneficial bacteria populations, such as *bifidobacteria*, increase short‐chain fatty acid production and enhance the secretion of specific cytokines, ultimately decreasing the *Salmonella enteritidis* population. Interestingly, the efficiency of enzymes and gizzard enlargement can be enhanced by using coarser particle sizes of the main dietary ingredients (Melo Durán et al. 2020). Increasing the size of the gizzard can prevent the entry of pathogenic bacteria into the small intestine, promote gizzard contractions, increase HCl secretion and retention time of the digesta in the gizzard and increase pepsin activity, resulting in improved protein digestion rates (Melo Durán et al. 2020). Researchers have noted that considering ingredient particle size, the mash form of a diet has a more significant effect on the development of the digestive tract in birds (Zaefarian et al. 2016). However, one disadvantage of pelleting is that all feed components, including heat‐sensitive additives, are exposed to hot steam. If heat‐sensitive additives are not adequately coated, their structure may be compromised, leading to reduced activity or even the occurrence of the Maillard reaction. Additionally, in high‐altitude locations with inefficient ventilation, the use of pelleted feed may contribute to metabolic diseases such as sudden death syndrome (SDS) and ascites. In such cases, processing corn for use in mash diets may be beneficial. Additional disadvantages of pelleted diets include the inability to adjust diets during disease outbreaks. Furthermore, one of the significant costs associated with pellet production is electricity and the depreciation of dies and rollers. Feeding mash diets with processed corn may avoid the disadvantages linked to pelleted diets. Therefore, alternatives for improving nutrient utilisation and mitigating potential negative impacts of pelleted feed may include thermal processing of the main ingredients or the supplementation of exogenous enzymes to mash diets. The objectives of the present study were to determine the effects of different conditioning temperatures of corn used in mash diets and the effects of adding exogenous enzymes to processed or unprocessed corn on the apparent metabolisable energy (AME) of corn, nutrient utilisation, growth performance, jejunum morphology and ileum microbial population in broiler chicks during the finisher period.

## 2 Materials and Methods

### 2.1 Diets and Treatments

Corn grains were ground using a hammer mill (650 mm rotor diameter, 100 HP, 75 kW, 1480 rpm with 64 hammers) and then sieved with a screen size of 5.0 mm. For the dietary treatments, ground corn was divided into four sections: one unconditioned and three conditioned separately at temperatures of 55°C, 70°C and 85°C for 150 s using a super‐conditioner (Dordaneh Livestock and Poultry Feed Co, Chenaran, Iran). The temperature of the conditioned corn was measured immediately after leaving the conditioner with an infrared thermometer (model Benetech GM 320, −50°C to 380°C), recording temperatures of 51°C, 67°C and 83°C for the conditioning temperatures of 55°C, 70°C and 85°C, respectively. The conditioner was capable of processing 1400 kg of feed per hour, featuring two shafts, each equipped with 16 paddles set at a 45° angle. The steam pressure and temperature used in this conditioner were 2 bars and 75°C, respectively, with steam introduced through three injection valves for improved and uniform distribution at the beginning, middle and end of the conditioning process.

After heat processing, the ground corn was dried at 45°C and then cooled at 25°C for 8 min. Then, it was bagged, labelled and sent to the rearing house for inclusion in the experimental diets. The experimental diets were prepared as mash based on corn and soybean meal (Table 1). Half of the experimental diets (four out of eight) were supplemented with 0.5 g/kg of a multi‐enzyme containing cellulases (6400 U), β‐glucanase (2000 U) and xylanase (22,000 U/g) (Rovabio, Adisseo, France). Notably, in this experiment, before the feed ingredients were mixed, only corn was processed before mixing the feed ingredients to reduce overall processing costs and to investigate the effects of single ingredient processing on the performance of broiler chicks. The exogenous enzyme was added to the diet after processing the corn to avoid exposure to heat.

**TABLE 1:** Ingredients and nutrient composition of experimental diets, *as‐fed basis*.

Ingredients (%)	Diet for determination of nutrient utilisation (Trial 1)	Finisher (25–42 days)
Corn (8.4% CP)^a^	96.23	59.24
Soybean meal (44% CP)	—	32.28
Soy oil	—	5.94
Calcium carbonate	0.94	0.91
Dicalcium phosphate	1.87	0.15
Vitamin premix^b^	0.25	0.25
Mineral premix^c^	0.25	0.25
dl‐Methionine	—	0.29
l‐Lysine HCl	—	0.19
l‐Threonine	—	0.06
Common salt	0.27	0.29
Sodium bicarbonate	0.12	0.12
Choline chloride (600 g/kg)	0.07	0.03
Calculated analysis (%)		
Metabolisable energy (kcal/kg)	3223	3200
Crude protein	8.18	19.50
Calcium	0.80	0.79
Available phosphorus	0.39	0.39
Methionine	0.17	0.59
Methionine + Cystine	0.34	0.91
Lysine	0.25	0.11
Threonine	0.28	0.88
Sodium	0.16	0.16
Chloride	0.21	0.25
DCAB (mEq/kg)	89.85	236.00
Analysed values (%)		
Dry matter	90.60	90.80
Crude protein (nitrogen × 6.25)	8.14	19.50
Calcium	0.77	0.83
Total phosphorus	0.51	0.57

Abbreviation: DCAB, dietary cation‒anion balance.

^a^ Unprocessed and processed ground corns at temperatures of 55°C, 70°C and 85°C for 150 s with or without Rovabio enzyme were used to prepare diets in the form of mash.

^b^ Vitamin premix provided per kg of diet: vitamin A (retinyl acetate), 15,000 U; vitamin D3, 5000 U; vitamin E (dl‐α‐tocopheryl acetate), 80 mg; vitamin K, 5 mg; thiamin, 3 mg; riboflavin,10 mg; pyridoxine, 5 mg; vitamin B_12_, 0.02 mg; niacin, 70 mg; choline chloride, 1800 mg; folic acid, 2 mg; biotin, 0.4 mg; pantothenic acid, 20 mg.

^c^ Mineral premix provided per kg of diet: Mn (manganese sulphate), 100 mg; Zn (zinc sulphate), 65 mg; Cu (copper sulphate), 5 mg; Se (sodium selenite), 0.22 mg; I (calcium iodate), 0.5 mg; and cobalt, 0.5 mg.

### 2.2 Birds and Management

In Experiment 1, the AME and apparent total tract retention (ATTR) of corn N, DM, Ca and TP were determined in basal diets where corn was the sole source of ME and N (Zaghari et al. 2020; Schiavone et al. 2017). The basal diets contained heat‐processed corn (at temperatures of 55°C, 70°C and 85°C) or unprocessed corn with or without enzyme supplementation at 0 and 0.5 g/kg diet. Total excreta were collected from 96 male broilers (Ross 308) across 8 treatments, with 6 replicates and 2 birds each from 26 to 28 days of age, housed in 48 battery cages. On Day 23, two male birds were selected from each experimental unit and randomly allocated to battery cages (1.0 × 1.0 × 0.5 m^3^; *L* × *W* × *H*). A 3‐day adaptation period was allowed for the birds before excreta collection began on Day 26. Birds were not fed for approximately 8 h before excreta collection, which occurred for 8 h after the allocated feed was consumed. Feed intake was recorded from Days 26–28. Excreta from each replicate cage were collected twice daily. After removing feathers, feed residues and other contaminants, excreta were dried in an oven at 55°C for 72 h. The total excreta were weighed and homogenised, and approximately 30% of the total excreta were randomly isolated, air‐dried, ground and stored at −20°C for further analysis. The dry matter, Ca, TP and N contents of the feed and excreta were measured according to AOAC (1990) methods. Gross energy was determined using an adiabatic calorimetric bomb (1281, PARR Instruments, USA). The AME values were calculated according to Zaghari et al. (2020).

In the second experiment, 480 broilers (Ross 308) were reared under standard conditions until 24 days of age and fed a practical diet of unprocessed corn and soybean meal. On Day 25, broilers with a mean body weight (BW) of 903 ± 20 g were fed experimental mash diets containing conditioned or unconditioned corn, with or without enzymes, until 42 days of age. A completely randomised design with a 4 × 2 factorial arrangement was employed to study the effects of conditioning temperatures of corn used in mash diets (unconditioned and conditioned at 55°C, 70°C and 85°C) and enzyme supplementation (0 or 0.5 g/kg diet). Six floor pens, each measuring 1.2 × 1.2 × 0.8 m^3^ (*L* × *W* × *H*) and covered with wood shavings (1.5 kg/m^2^), were designated for each treatment. Each pen housed 10 birds (5 males and 5 females). The experimental mash diet for the finisher phase was formulated according to the Ross 308 strain guidelines (Aviagen 2014; Table 1) and provided to the birds ad libitum along with drinking water. Each experimental unit included a trough feeder and two water nipples. The house temperature, relative humidity and lighting programme were maintained at 21°C ± 2°C, 65% and an 18L:6D photoperiod, respectively, from Days 25 to 42.

### 2.3 Growth Performance

The birds in each experimental unit were weighed on Days 25 and 42. Average daily gain (ADG) in each replicate was calculated by subtracting the average initial weight of the group from the average final weight and dividing by the number of days the experiment lasted. Average daily feed intake (ADFI) was calculated by subtracting the feed remaining from the feed provided in each pen and dividing by the number of days the experiment lasted. Feed conversion ratio (FCR) was calculated as ADFI divided by ADG and adjusted for mortality (Dersjant‐Li et al. 2020). The European production efficiency factor (EPEF) was calculated according to Kryeziu et al. (2018).

### 2.4 Carcass Yield

On Day 42, two male birds were selected from each replicate, fasted for 6 h and euthanised under deep anaesthesia via intravenous injection of pentobarbital sodium. After skin removal, the carcass weight, breast yield, thigh weight, back and neck, wing weight and internal organs were separated and weighed. Additionally, the lengths of different sections of the small intestine (duodenum, jejunum and ileum) were measured.

### 2.5 Intestinal Morphology

Two male broilers were selected from each replicate on Day 42. The birds were euthanised under deep anaesthesia via intravenous injection of pentobarbital sodium. Approximately 1 cm sections from the midpoint of the jejunum (from the duodenal loop to Meckel diverticulum) were collected, flushed with 0.9% saline and fixed in 10% buffered formalin. The tissue samples were removed from the solution, dehydrated through a series of graded ethanol solutions, cleared in xylene and infiltrated with paraffin. The samples were embedded in paraffin blocks and sectioned at 5–6 µm thickness using a rotating microtome. The sections were floated in 40°C distilled water and placed on glass slides after straightening any wrinkles. The slides were then placed on a warm plate (45°C), allowing any excess paraffin to melt while drying. The slides were stained with hematoxylin and eosin. All chemicals were obtained from Sigma‐Aldrich Co. (St. Louis, MO). Micrographs were captured using an Olympus BX41 optical microscope (Olympus Corporation, Tokyo, Japan) fitted with a digital video camera. The images were analysed using image software. Morphometric measurements were performed on 10 intact villi from each sample. The morphometric indices included villus height (from the tip of the villus to the crypt), villus width (the average width at one‐third and two‐thirds of the villus) and crypt depth (from the base of the villus to the submucosa; Teymouri and Hassanabadi 2021).

### 2.6 Microbial Analysis

On Day 42, two male birds from each replicate were selected and euthanised under deep anaesthesia via intravenous injection of pentobarbital sodium. Approximately 3 g of ileal digesta was collected into sterile tubes. To assess microbial counts, 1 g of the sample was used to prepare serial 10‐fold dilutions in buffered peptone water. Lactobacillus bacteria were enumerated using de Man, Rogosa and Sharpe (MRS; Merck, Germany) agar as well as Rogosa agar (Al‐Baadani et al. 2022). Salmonella and coliform bacteria were assessed on brilliant‐green phenol‐red lactose sucrose (BPLS) agar and violet red bile (VRB; Merck, Germany) agar, respectively (Hu et al. 2021; Cetin et al. 2016). The plates were incubated anaerobically at 37°C for 24 h.

### 2.7 Statistical Analysis

A completely randomised design with a 4 × 2 factorial arrangement was employed in both experiments. The main factors included corn conditioning temperatures (unconditioned and conditioned at 55°C, 70°C and 85°C) and levels of enzyme supplementation (0 and 0.5 g/kg diet). Prior to data analysis, normality and homoscedasticity were evaluated using the Shapiro–Wilk test (Shapiro and Wilk 1965). All data were analysed using the general linear model (GLM) procedure in SAS software (version 9.4; Statistical Analysis System Institute 2012), and differences between means were assessed with Tukey's post hoc test (*p* ≤ 0.05).

## 3 Results

### 3.1 Nutrient Utilisation

The effects of different conditioning temperatures and enzyme supplementation on the AME and AMEn values, as well as DM, CP, Ca, TP utilisation in broilers from 26 to 28 days of age, are presented in Table 2. The AME value of corn thermally processed at 85°C was significantly higher compared to corn processed at 55°C and unprocessed corn. Furthermore, thermal processing of corn at 55°C resulted in greater Ca utilisation than the unprocessed conditions (*p* < 0.05). Enzyme supplementation did not significantly affect the AMEn value of corn. Neither thermal processing nor enzyme supplementation significantly influenced the AMEn value or the ATTR of corn DM, CP, Ca or TP. No significant interactions were found between corn conditioning and enzyme supplementation regarding the AMEn values or nutrient utilisation in broiler chicks. Orthogonal contrast analyses indicated that thermal processing of corn significantly increased (*p* < 0.01) AME and AMEn values compared with unconditioned corn in broiler chicks aged 26–28 days (3192.5 vs. 3144.3 and 3174.7 vs. 3143.7 kcal/kg, respectively). When comparing processed corn at different temperatures without enzyme supplementation to unprocessed corn, the AME and AMEn values of the processed corn were significantly greater (*p* < 0.05) (3179.5 vs. 3116.3 and 3172.5 vs. 3114.1 kcal/kg, respectively). Additionally, when comparing enzyme‐supplemented processed corn to enzyme‐supplemented unprocessed corn, the AME value of processed corn was significantly higher (*p* < 0.05) (3205.5 vs. 3172.2 and 3176.9 vs. 3168.4 kcal/kg, respectively; Table 2). Corn heat processing also led to increased ATTR for Ca in broilers during the finisher period (*p* < 0.05). However, orthogonal comparisons for DM, CP or TP utilisation were not statistically significant (*p* > 0.05).

**TABLE 2:** Effect of corn conditioning and enzyme supplementation on apparent metabolisable energy, total tract apparent retention of crude protein and dry matter in broiler chicks during 26–28 days.^1^

Treatments		Apparent metabolisable energy (kcal/kg)	Metabolisable energy, corrected for N (kcal/kg)	Crude protein (%)	Dry matter (%)	Calcium (%)	Total phosphorus (%)
Main effects							
Temperature^2^							
Unprocessed		3144.3^b^	3143.7	44.5	80.55	0.41^b^	0.49
55°C		3129.0^b^	3124.8	38.2	80.54	0.56^a^	0.57
70°C		3188.4^a,b^	3184.7	36.8	79.55	0.48^a,b^	0.52
85°C		3260.2^a^	3214.7	38.3	81.19	0.49^a,b^	0.49
SE		27.61	34.44	0.02	0.007	0.029	0.27
Enzyme^3^							
−		3163.7	3159.8	38.1	80.92	0.49	0.52
+		3197.2	3174.7	40.8	79.99	0.48	0.51
SE		20.01	23.79	0.01	0.005	0.021	0.019
Interaction effects							
Enzyme × processing							
−	Unprocessed	3116.3	3114.1	42.6	80.8	0.42	0.50
	55°C	3133.3	3129.1	38.0	81.7	0.56	0.57
	70°C	3203.6	3200.1	35.1	80.7	0.49	0.54
	85°C	3201.7	3188.4	36.5	80.4	0.50	0.50
+	Unprocessed	3172.2	3168.4	46.3	80.3	0.40	0.49
	55°C	3124.6	3120.4	38.3	79.4	0.56	0.57
	70°C	3173.1	3169.2	38.5	78.4	0.47	0.49
	85°C	3318.8	3240.9	40.0	82.0	0.48	0.48
	SE	39.05	46.44	0.03	0.01	0.42	0.039
					*p* values		
Processing		0.01	0.22	0.12	0.48	0.01	0.19
Enzyme		0.24	0.61	0.26	0.22	0.63	0.53
Processing × enzyme		0.27	0.73	0.94	0.23	0.98	0.93
Orthogonal contrast analysis							
P vs. Unp.		0.003	0.0007	0.21	0.06	0.004	0.332
P–E vs. Unp–E		0.03	0.01	0.31	0.23	0.05	0.45
P + E vs. Unp + E		0.04	0.01	0.49	0.12	0.05	0

*Note*: Means without common superscript (a, b) within a column are significantly different (*p* < 0.05). −E: without enzyme supplementation; +E: with enzyme supplementation.

Abbreviations: P, processed; SE, standard error of the mean; Unp, unprocessed.

^1^ Each value represents the mean of six replicates (two birds per replicate).

^2^ Unprocessed and processed ground corns at temperatures of 55°C, 70°C and 85°C for 150 s were included in the mash diets.

^3^ Rovabio enzyme was provided from Adisseo Company (France) and supplemented at the rate of 0.50 g/kg diet.

### 3.2 Growth Performance

The main effect of corn conditioning had no significant impact on BW at 42 days, WG, FI or FCR of broilers from 25 to 42 days (Table 3). There were no significant interactions between corn processing and enzyme supplementation for BW at 42 days, WG, or FI from 25 to 42 days (Table 3). Corn processed at 55°C without enzyme supplementation and corn processed at 70°C with enzyme supplementation exhibited the lowest FCR (*p* < 0.05). Orthogonal comparisons of growth performance from 25 to 42 days revealed that birds fed diets containing processed corn had significantly greater feed intake (160.7 vs. 150.4 g/bird/d; *p* < 0.05) compared to those fed unprocessed corn. Likewise, birds fed processed corn without enzyme supplementation had significantly higher FI (167.5 vs. 152.5 g/bird/d, *p* < 0.01) than those fed unprocessed corn without enzyme supplementation. There were no significant differences in FI among birds fed processed corn in diets supplemented with exogenous enzymes compared to those in enzyme‐supplemented diets containing unprocessed corn. No significant differences (*p* > 0.05) were observed for BW, WG or FCR (Table 3). The main effect of enzyme supplementation on the EPEF was significant, with the addition of the enzyme leading to a higher EPEF (*p* < 0.05). However, the main effects of heat processing temperature and the interactions between enzyme addition and heat processing temperature on EPEF were not significant (*p* > 0.05).

**TABLE 3:** Effect of corn conditioning and enzyme supplementation on the growth performance of broilers (25–42 days).^1^

Treatments		Body weight (g/bird)	Weight gain (g/bird/day)	Feed intake (g/bird/day)	Feed conversion ratio (g/g)	EPEF
Main effects						
Temperature^2^						
Unprocessed		2548.9	89.3	150.4	1.74^b^	285.90
55°C		2539.7	93.0	158.7	1.75^b^	278.02
70°C		2544.2	93.8	163.5	1.74^b^	279.11
85°C		2531.0	87.9	160.0	1.83^a^	259.31
SE		46.49	2.85	3.90	0.02	13.52
Enzyme^3^						
−		2570.5	93.3	162.0^a^	1.74	261.44^b^
+		2511.4	88.7	154.3^b^	1.79	289.74^a^
SE		32.87	2.01	2.76	0.02	9.56
Interaction effects						
Enzyme × processing						
−	Unprocessed	2535.9	89.2	152.5	1.73^ab^	289.44
	55°C	2593.0	96.1	160.5	1.68^b^	254.65
	70°C	2568.5	96.6	174.4	1.80^a^	271.27
	85°C	2584.7	91.3	160.6	1.77^ab^	230.87
+	Unprocessed	2562.0	89.4	148.3	1.74^ab^	281.64
	55°C	2486.4	90.0	157.0	1.82^a^	297.48
	70°C	2519.9	91.0	152.6	1.69^b^	285.63
	85°C	2477.3	84.5	159.4	1.90^a^	293.44
	SE	68.67	4.21	5.26	0.14	19.98
				*p* value		
Processing		0.99	0.39	0.12	0.05	0.668
Enzyme		0.21	0.11	0.05	0.08	0.0458
Processing × enzyme		0.71	0.80	0.23	0.05	0.285
Orthogonal contrast analysis						
P vs. Unp.		0.83	0.48	0.002	0.36	0.154
P–E vs. Unp–E		0.40	0.16	0.002	0.67	0.498
P + E vs. Unp + E		0.59	0.76	0.20	0.35	0.395

*Note*: Means without common superscript (a, b) within a column are significantly different (*p* < 0.05). −E: without enzyme supplementation; +E: with enzyme supplementation.

Abbreviations: P, processed; SE, standard error of the mean; Unp, unprocessed.

^1^ Each value represents the mean of 6 replicates (10 birds per replicate).

^2^ Unprocessed and processed ground corns at temperatures of 55°C, 70°C and 85°C for 150 s were included in the mash diets.

^3^ Rovabio enzyme was provided from Adisseo Company (France) and supplemented at the rate of 0.50 g/kg diet.

### 3.3 Carcass Traits and Intestinal Morphology

The effects of corn thermal processing and enzyme supplementation on the relative weights of carcasses and body organs of broilers at 42 days of age are shown in Table 4. Neither corn conditioning nor enzyme supplementation, nor their interactions, significantly affected the relative weights of the edible carcass, thighs, breast yield, neck + back, liver, pancreas, heart, spleen, bursa of Fabricius or abdominal fat. Orthogonal comparisons indicated that broilers fed processed corn without enzyme supplementation had significantly lower relative weights of the liver, pancreas and bursa of Fabricius compared to those fed unprocessed corn without enzyme supplementation (0.22% vs. 0.30%; 0.34% vs. 0.40%; and 0.61% vs. 0.73% of live weight, respectively; *p* < 0.05). Corn conditioning, dietary enzyme supplementation and their interaction did not significantly affect the relative weights of gut parts or the length of the small intestine, except for ileum weight. The relative weight of the ileum in non‐enzyme‐supplemented diets decreased with increasing processing temperature, whereas the opposite trend was observed in enzyme‐supplemented diets (*p* < 0.05). Orthogonal comparisons revealed that, compared to unprocessed corn, birds fed diets containing heat‐conditioned corn without enzyme supplementation had significantly lower relative weights of the gizzard, duodenum and ileum (0.58% vs. 0.47%; 1.34% vs. 1.19%; and 1.24% vs. 1.44% of live weight, respectively; *p* < 0.05).

**TABLE 4:** Effect of corn conditioning and enzyme supplementation on carcass characteristics (% of live body weight) in broiler chickens (42 days).^1^

Treatments		Edible carcass	Thighs	Breast yield	Neck + back	Liver	Pancreas	Heart	Spleen	Bursa of Fabricius	Abdominal fat pad
Main effects											
Temperature^2^											
Unprocessed		2.26	18.1	26.3	19.9	0.26	0.37	0.10	0.15	0.66	0.012
55°C		2.32	18.2	26.5	20.5	0.22	0.35	0.10	0.15	0.55	0.013
70°C		2.30	18.6	25.6	20.7	0.25	0.32	0.14	0.17	0.62	0.012
85°C		2.37	18.9	26.8	20.9	0.23	0.37	0.12	0.15	0.59	0.010
SE		0.08	0.49	0.88	0.39	0.013	0.015	0.009	0.016	0.031	0.001
Enzyme^3^											
−		2.30	18.5	25.91	20.79	0.25	0.35	0.12	0.16	0.64^a^	0.012
+		2.33	18.4	26.65	20.27	0.23	0.34	0.11	0.15	0.57^b^	0.011
SE		0.06	0.35	0.62	0.27	0.01	0.01	0.01	0.01	0.02	0.001
Interaction effects											
Enzyme × processing											
−	Unprocessed	2.19	18.0	26.2	20.2	0.30	0.40	0.11	0.15	0.73	0.010
	55°C	2.27	18.4	26.4	20.1	0.22	0.34	0.10	0.16	0.60	0.017
	70°C	2.29	18.3	23.4	21.0	0.25	0.33	0.12	0.17	0.64	0.012
	85°C	2.44	19.2	27.7	22.0	0.24	0.35	0.14	0.17	0.60	0.010
+	Unprocessed	2.32	18.1	26.4	19.6	0.22	0.33	0.10	0.16	0.59	0.014
	55°C	2.37	18.1	26.5	20.9	0.23	0.35	0.09	0.14	0.51	0.009
	70°C	2.30	18.8	27.8	20.5	0.25	0.31	0.16	0.16	0.61	0.013
	85°C	2.31	18.6	25.9	20.0	0.21	0.39	0.11	0.13	0.58	0.010
SE		0.12	0.70	1.25	0.55	0.019	0.02	0.13	0.023	0.044	0.002
											*p* value
Processing		0.83	0.93	0.78	0.30	0.27	0.09	0.06	0.86	0.11	0.55
Enzyme		0.75	0.66	0.41	0.19	0.08	0.52	0.73	0.47	0.05	0.62
Processing × enzyme		0.75	0.88	0.11	0.14	0.11	0.08	0.10	0.74	0.50	0.06
Orthogonal contrast analysis											
P vs. Unp.		0.44	0.36	0.97	0.09	0.17	0.27	0.20	0.90	0.06	0.20
P–E vs. Unp–E		0.39	0.58	0.85	0.38	0.01	0.03	0.32	0.20	0.04	0.35
P + E vs. Unp + E		0.93	0.33	0.77	0.07	0.56	0.40	0.41	0.65	0.56	0.004

*Note*: Means without common superscript (a, b) within a column are significantly different (*p* < 0.05). −E: without enzyme supplementation; +E: with enzyme supplementation.

Abbreviations: P, processed; SE, standard error of the mean; Unp, unprocessed.

^1^ Each value represents the mean of six replicates.

^2^ Unprocessed and processed ground corns at temperatures of 55°C, 70°C and 85°C for 150 s were included in the mash diets.

^3^ Rovabio enzyme was provided from Adisseo Company (France) and supplemented at the rate of 0.50 g/kg diet.

**FIGURE 1:**
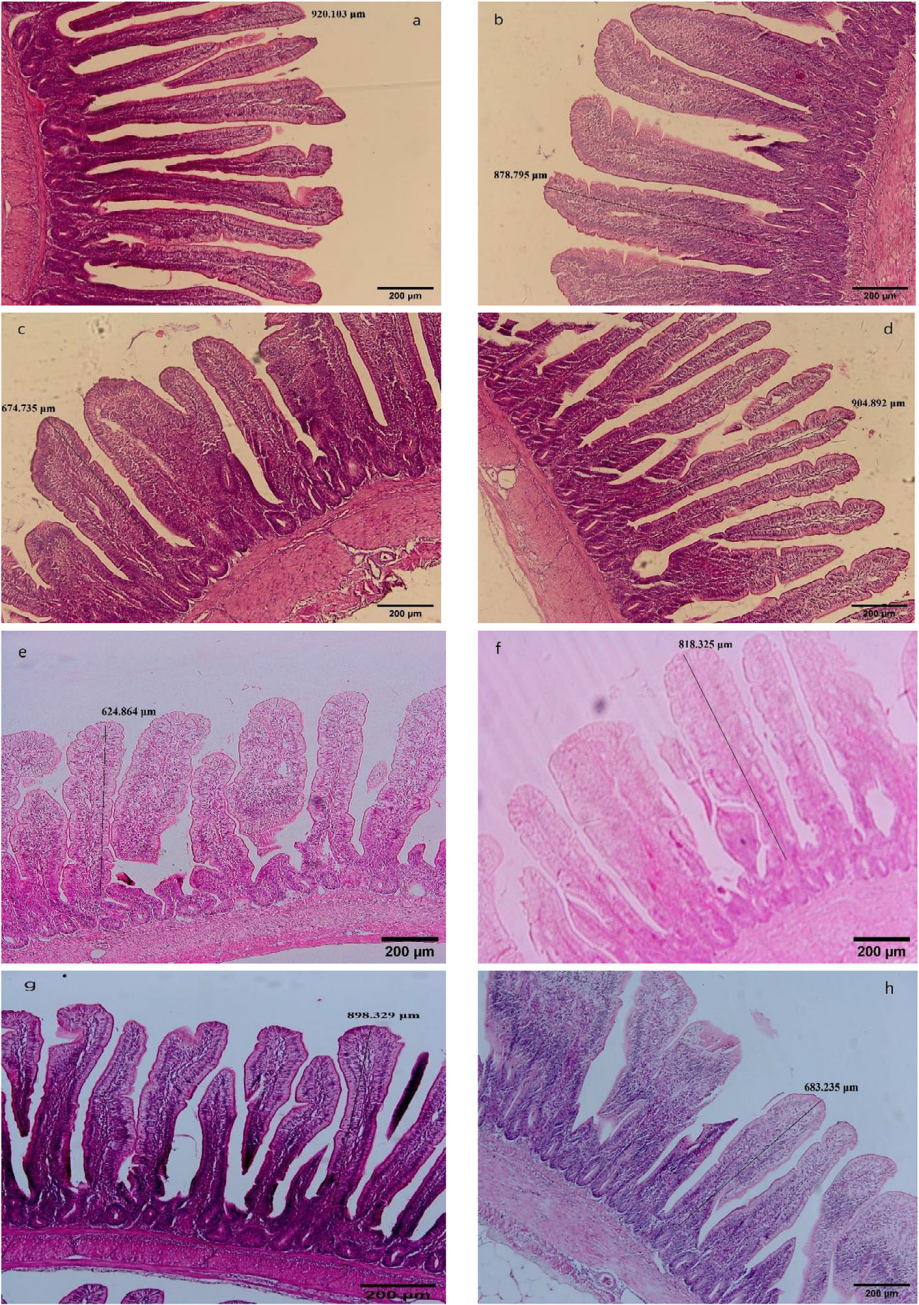
Histomorphometric analysis of the jejunum of 42 day‐old broilers. Villus height in treatments: without corn processing (a); corn processing at 55°C (b); corn processing at 55°C (c); corn processing at 70°C (d); corn processing at 85°C (e); without corn processing + enzyme (f); corn processing at 55°C + enzyme (g); processing at 70°C + enzyme; corn processing at 85°C + enzyme (h). Images were taken at 400× magnification. The scale bar represents 200 µm.

The effects of corn conditioning and enzyme supplementation on gut morphology and histomorphology in broiler chicks at 42 days are presented in Tables 5 and 6, respectively. Light microscopy images of the jejunal histomorphology for the different groups of broilers are shown in Figure 1.

**TABLE 5:** Effect of corn conditioning and enzyme supplementation on gut morphology of broiler chickens at 42 days.^1^

Treatments		% of live body weight					m/kg body weight			
		Proventriculus	Gizzard	Duodenum	Jejunum	Ileum	Small intestine	Duodenum	Jejunum	Ileum
Main effects										
Temperature^2^										
Unprocessed		1.45	0.54	1.29	0.91	1.34	224.5	37.4	91.7	95.4
55°C		1.35	0.46	1.25	0.85	1.23	220.4	36.2	88.4	95.8
70°C		1.30	0.50	1.21	0.84	1.30	222.1	37.1	87.5	97.5
85°C		1.44	0.50	1.22	0.79	1.28	231.2	37.5	93.9	99.8
SE		0.05	0.021	0.036	0.033	0.034	5.26	0.79	2.08	3.08
Enzyme^3^										
−		1.43	0.50	1.23	0.84	1.28	223.9	37.8	89.3	96.84
+		1.33	0.50	1.26	0.85	1.29	225.2	36.3	91.5	97.37
SE		0.036	0.014	0.025	0.024	0.024	3.71	0.56	1.47	2.18
Interaction effects										
Enzyme × processing										
−	Unprocessed	1.56	0.58	1.34	0.93	1.41^a^	224.1	38.4	91.5	94.3
	55°C	1.36	0.44	1.24	0.84	1.26^bc^	217.4	37.1	85.8	94.5
	70°C	1.27	0.48	1.12	0.79	1.24^bc^	223.9	39.0	86.8	98.1
	85°C	1.55	0.50	1.21	0.82	1.23^bc^	230.4	36.8	93.1	100.5
+	Unprocessed	1.35	0.50	1.24	0.89	1.28^abc^	224.9	36.5	91.9	96.5
	55°C	1.34	0.48	1.27	0.87	1.20^c^	223.4	35.3	91.1	97.0
	70°C	1.33	0.53	1.29	0.88	1.36^ab^	220.4	35.3	88.3	96.9
	85°C	1.32	0.50	1.22	0.77	1.33^abc^	232.0	38.3	94.6	99.1
SE		0.07	0.029	0.051	0.47	0.048	7.43	1.12	2.94	4.36
										*p* value
Processing		0.13	0.13	0.39	0.13	0.17	0.49	0.63	0.14	0.73
Enzyme		0.06	0.93	0.42	0.81	0.82	0.81	0.07	0.30	0.86
Processing × enzyme		0.13	0.13	0.08	0.50	0.03	0.93	0.15	0.84	0.94
Orthogonal contrast analysis										
P vs. Unp.		0.16	0.05	0.17	0.25	0.15	0.99	0.61	0.48	0.48
P–E vs. Unp–E		0.08	0.01	0.05	0.22	0.05	0.97	0.51	0.43	0.53
P + E vs. Unp + E		0.83	0.82	0.97	0.71	0.90	0.96	0.87	0.87	0.77

*Note*: Means without common superscript (a, b) within a column are significantly different (*p* < 0.05). −E: without enzyme supplementation; +E: with enzyme supplementation.

Abbreviations: P, processed; SE, standard error of the mean; Unp, unprocessed.

^1^ Each value represents the mean of six replicates.

^2^ Unprocessed and processed ground corns at temperatures of 55°C, 70°C and 85°C for 150 s were included in the mash diets.

^3^ Rovabio enzyme was provided from Adisseo Company (France) and supplemented at the rate of 0.50 g/kg diet.

**TABLE 6:** Effect of corn conditioning and enzyme supplementation on jejunum histomorphology of broiler chickens at 42 days.^1^

Treatments		Villus height (µm)	Villus width (µm)	Crypt depth (µm)	Villus height to crypt depth
Main effects					
Temperature^2^					
Unprocessed		796.0^b^	171.8	187.0	4.28
55°C		893.2^a^	154.5	179.0	5.12
70°C		935.5^a^	174.8	185.5	5.15
85°C		932.5^a^	175.8	196.5	4.76
SE		18.11	7.39	8.72	0.24
Enzyme^3^					
−		887.1	164.5	187.4	4.81
+		891.5	173.9	186.6	4.85
SE		12.80	5.22	6.17	0.17
Interaction effects					
Enzyme × processing					
−	Unprocessed	743.0^c^	174.0	191.5	3.87
	55°C	923.5^ab^	140.0	172.5	5.55
	70°C	959.0^a^	173.0	196.0	4.92
	85°C	923.0^ab^	171.0	189.5	4.90
+	Unprocessed	849.0^b^	169.5	182.5	4.69
	55°C	862.9^b^	169.0	185.5	4.69
	70°C	912.0^ab^	176.5	175.0	5.38
	85°C	942.0^a^	180.5	203.5	4.63
	SE	25.61	10.45	12.34	0.35
					*p* value
Processing		0.0001	0.17	0.57	0.07
Enzyme		0.81	0.21	0.93	0.88
Processing × enzyme		0.013	0.44	0.42	0.10
Orthogonal contrast analysis					
P vs. Unp.		0.0001	0.70	0.97	0.02
P–E vs. Unp–E		0.0001	0.39	0.69	0.008
P + E vs. Unp + E		0.14	0.58	0.72	0.61

*Note*: Means without common superscript (a, b) within a column are significantly different (*p* < 0.05). −E: without enzyme supplementation; +E: with enzyme supplementation.

Abbreviations: P, processed; SE, standard error of the mean; Unp, unprocessed.

^1^ Each value represents the mean of six replicates.

^2^ Unprocessed and processed ground corns at temperatures of 55°C, 70°C and 85°C for 150 s were included in the mash diets.

^3^ Rovabio enzyme was provided from Adisseo Company (France) and supplemented at the rate of 0.50 g/kg diet.

Thermal processing had a significant effect (*p* < 0.05) on villus height, with broilers fed heat‐conditioned corn (55°C, 70°C or 85°C) exhibiting greater jejunal villus heights compared to those fed unprocessed corn (Figure 1). The interactions between thermal processing and enzyme addition were also significant (*p* < 0.05). Birds fed diets containing unprocessed corn without enzymes had the lowest villus height (743 µm) among the groups. Orthogonal analysis showed that corn processing increased jejunal villus height and the ratio of villus height to crypt depth (*p* < 0.05). Compared to birds fed heat‐processed corn without enzyme supplementation, those fed unprocessed corn without enzyme supplementation had lower villus height and villus height to crypt depth ratios in their jejunum (743 vs. 935.16; 3.87 vs. 5.12; *p* < 0.05).

### 3.4 Microbial Analysis

The effects of corn conditioning and enzyme supplementation on microbial counts (Log_10_ CFU/g) of ileal contents in broiler chicks at 42 days are presented in Table 7. Feeding heat‐processed corn at 55°C resulted in the lowest *Clostridium* count in the ileal digesta (*p* < 0.05). No significant differences were observed among the microbial counts of Lactobacillus, *Bifidobacterium*, *Clostridium*, *E. coli* and Salmonella in the ileal contents of broiler chicks at 42 days. Orthogonal comparisons did not reveal significant differences among the microbial populations in the ileal digesta of the broilers (*p* > 0.05).

**TABLE 7:** Effect of corn conditioning and enzyme supplementation on microbial count (Log_10_ CFU/g) of ileum contents in broiler chickens at 42 days.^1^

Treatments		*Lactobacillus*	*Bifidobacterium*	*Clostridium*	*Escherichia coli*	*Salmonella*
Main effects						
Temperature^2^						
Unprocessed		7.39	5.95	1.61^a^	4.91	Not find
55°C		8.36	6.35	1.20^b^	4.63	Not find
70°C		8.30	6.45	1.60^a^	4.92	Not find
85°C		8.25	6.54	1.68^a^	4.99	Not find
SE		0.30	0.27	0.10	0.39	
Enzyme^3^						
−		8.13	6.44	1.50	4.75	Not find
+		8.02	6.20	1.54	4.97	Not find
SE		0.21	0.19	0.072	0.28	
Interaction effects						
Enzyme × processing						
−	Unprocessed	7.81	6.16	1.63	4.82	Not find
	55°C	8.47	6.77	1.20	4.49	Not find
	70°C	8.18	6.48	1.73	4.51	Not find
	85°C	8.08	6.37	1.46	5.19	Not find
+	Unprocessed	6.98	5.75	1.60	4.99	Not find
	55°C	8.26	5.94	1.20	4.76	Not find
	70°C	8.42	6.42	1.46	5.34	Not find
	85°C	8.43	6.71	1.90	4.80	Not find
SE		0.43	0.38	0.14	0.56	
					*p* value	
Processing		0.12	0.46	0.01	0.91	
Enzyme		0.72	0.39	0.74	0.58	
Processing × enzyme		0.53	0.50	0.15	0.76	
Orthogonal contrast analysis						
P vs. Unp.		0.14	0.37	0.68	0.82	
P–E vs. Unp–E		0.87	0.73	0.62	0.66	
P + E vs. Unp + E		0.08	0.38	0.91	0.91	

*Note*: Means without common superscript (a, b) within a column are significantly different (*p* < 0.05). −E: without enzyme supplementation; +E: with enzyme supplementation.

Abbreviations: P, processed; SE, standard error of the mean; Unp, unprocessed.

^1^ Each value represents the mean of six replicates.

^2^ Unprocessed and processed ground corns at temperatures of 55°C, 70°C and 85°C for 150 s were included in the mash diets.

^3^ Rovabio enzyme was provided from Adisseo Company (France) and supplemented at the rate of 0.50 g/kg diet.

## 4 Discussion

### 4.1 Nutrient Utilisation

Thermal processing can change the structure of cereal starch, protein and fibre, positively impacting the accessibility of digestive enzymes to nutrients and increasing their digestibility (Østergård et al. 1989). In this study, thermal processing of corn at 85°C significantly increased the AME value compared to both heat conditioning at 55°C and unprocessed corn. Additionally, thermal processing at 55°C improved Ca utilisation compared to unprocessed corn. In contrast, Abdollahi et al. (2020) reported a decrease in diet AME value by approximately 24 kcal/kg for every 10°C increase when conditioning temperatures ranged from 60°C to 90°C. These discrepancies may stem from variations in basal diet ingredients and conditioning temperatures. In the present study, corn was subjected to heat conditioning with steam at temperatures of 55°C, 70°C and 85°C. Parallel to the results of the present study, Jiménez‐Moreno et al. (2016) found that the AME value of heat‐processed corn exceeded that of unprocessed corn, likely due to the dissolution of hemicellulose, which enhances the uptake of simple sugars as energy sources. López et al. (2007) noted no differences in the coefficient of DM digestibility between mash and pellet diets. The digestibility of CP and amino acids is influenced by diet ingredients, heat temperature, feed form and the levels of disulphide bonds and sulfhydryl groups (Abdollahi et al. 2011; Selle et al. 2012). Mild temperatures and short‐term conditioning may not have beneficial effects on the digestibility of amino acids, whereas prolonged high temperatures can decrease amino acid availability due to the destruction of protein secondary structures (Amezcua and Parsons 2007). The development of indigestible starch‐protein and starch‐lipid complexes at high conditioning temperatures can hinder nutrient absorption (Netto et al. 2019). However, Scott et al. (1997) suggested that heat conditioning can break disulphide bridges in proteins, improving enzyme efficiency and protein digestibility. Nevertheless, protein digestibility may decrease with high temperatures and extended conditioning times (Massuquetto et al. 2020). Furthermore, heat treatment of corn–soybean meal diets has been shown not to alter the availability of phytate phosphorus in broilers (Edwards et al. 1999). Additionally, Kirkpinar and Basmacioglu (2006) reported that the concentrations of DM, Ca and P in broilers’ tibia were not influenced by the pelleting temperature. Thus, factors such as temperature, processing time, ingredient type and diet form significantly impact AME and nutrient utilisation in thermally processed feeds like corn.

### 4.2 Growth Performance

The current study found that feeding mash diets containing heat‐processed corn at 55°C without enzyme supplementation or corn processed at 70°C with enzyme supplementation resulted in the lowest FCR in broilers during the finisher period. Heat treatment likely increased the gelatinisation of corn starch, increased starch degradation by endogenous enzymes, increased fibre degradation and promoted exogenous enzyme activity (Vranjes and Wenk 1995). Orthogonal comparisons indicated that heat processing of corn significantly increased the FI of broilers. These findings contrast with Rueda et al. (2022), who reported no significant effect of varying heat processing temperatures on FI of broilers from 15 to 49 days. However, previous research has indicated that increasing conditioning temperatures from 60°C to 90°C (Abdollahi et al. 2010) or 75°C to 90°C can enhance broiler weight gain (Amerah et al. 2013). Conversely, Selle et al. (2013) reported that increasing conditioning temperatures from 65°C to 95°C increased FCR of broilers without affecting FI. Silversides and Bedford (1999) reported that heating wheat‐based diets above 80°C their nutritive value. High‐temperature conditioning can also increase feed and intestinal digesta viscosity, complicating the effects of heat treatment on broiler growth performance. These discrepancies may arise from variations in dietary ingredient proportions (e.g., fat, oil, wheat, corn and barley), non‐starch polysaccharide content, machinery used and processing parameters (Cowieson et al. 2005; Peisker 2006; Abdollahi et al. 2013; Liu et al. 2013). Previous studies show that adding dietary carbohydrases can break down NSP compounds, reducing viscosity and enhancing digestion rates (Zhang et al. 2014), thereby improving diet AME values (Nian et al. 2011). Cowieson et al. (2005) concluded that adding xylanases to high‐temperature processed diets was more effective than mild heat processing. They reported that xylanase supplementation was particularly effective in feeds processed at 85°C or 90°C compared to those processed at 80°C. Enzyme supplementation in diets conditioned at high temperatures (85°C) did not positively influence birds’ FCR (Gracia et al. 2003). The improvement in EPEF observed in the present study aligns with findings from Gilani et al. (2021), who reported that dietary supplementation with a combination of xylanase and beta‐glucanase increased EPEF in broilers fed corn–soybean meal‐based diets.

### 4.3 Carcass Traits and Intestinal Morphology

Multiple factors, including coccidiosis, stress, poor hygiene and immunosuppression, contribute to intestinal disorders; however, feed processing and enzyme supplementation may positively influence intestinal health, gizzard development and motility (Mateos et al. 2004). The main effects of corn conditioning and enzyme supplementation, as well as their interactions, did not significantly affect the relative weights of the edible carcass, thighs, breast yield, neck + back, liver, pancreas, heart, spleen, bursa of Fabricius or abdominal fat. Birds fed processed corn without enzyme supplementation showed significantly lower relative weights of the liver, pancreas, bursa of Fabricius, gizzard, duodenum and ileum compared to those fed unprocessed corn without enzyme supplementation. Similarly, Ghobadi and Karimi (2012) reported that feed processing and enzyme addition to wheat‐based diets did not significantly affect carcass characteristics, except for gizzard relative weight, which was reduced at 36 days due to decreased energy requirements for feed particle breakdown. Dessimoni et al. (2019) reported that adding xylanase and beta‐glucanase or a combination of both with phytase had no significant effect on carcass traits. Conversely, dos Santos et al. (2017) found that the combination of xylanase and phytase improved carcass and breast yields in broilers. These variations may result from differences in enzyme activities, dosages, substrate levels, bird age and strain and gut health status.

In the present study, corn heat processing increased jejunal villus height and the villus height‐to‐crypt depth ratio. In contrast, Salavati et al. (2021) reported that thermal conditioning of wheat decreased jejunal villus height compared to unprocessed wheat. Gracia et al. (2003) reported that heat processing and enzyme supplementation increased intestinal villus height in broilers. Villus height is a physiological marker of animal health in broilers (Shang et al. 2015). Recently, Alqhtani et al. (2022) reported that adding enzyme cocktails to diets increased intestinal villus height, enhancing gut absorption area. They suggested that enzyme cocktails mitigated the negative effects of NSP content on intestinal villi, leading to increased villus height. In this study, enzyme addition numerically increased jejunal villus height, but this difference was not statistically significant, possibly due to insufficient enzyme dosage to detect notable morphological changes.

### 4.4 Microbial Analysis

As previously mentioned, thermal processing of corn at 55°C decreased the *Clostridium* count in ileal digesta compared to the control group at 42 days; however, higher processing temperatures did not significantly affect *Clostridium* counts compared to control group. Additionally, neither thermal processing temperature nor enzyme supplementation affected Lactobacillus, *Bifidobacterium*, *E. coli* or Salmonella populations in the ileal contents. In contrast, Boroojeni et al. (2014a) reported that higher feed processing temperatures (85°C, 110°C or 130°C) increased Lactobacillus populations in the crop and ileum, although they had no significant effect on Clostridia or Enterobacteria populations. Previous studies reveal that heat conditioning of feed can cause molecular modifications that affect the affect gut bacterial counts (Hemetsberger et al. 2022). Notably, high conditioning temperatures can adversely affect gut health by increasing intestinal digesta viscosity due to NSP solubility (Cowieson et al. 2005), inactivating endogenous enzymes (Cowieson et al. 2005), promoting pathogenic bacteria growth and producing resistant starch (Abdollahi et al. 2011). The conditioning temperature, thermal processing time, feed ingredients, additives, bird age and strain can all directly impact intestinal microbial populations (Bedford and Cowieson 2012). Contrary to our findings, carbohydrase enzymes like xylanase can modify intestinal bacteria populations by breaking down NSPs and increasing short‐chain fatty acid production in the gut. Yaghobfar and Kalantar (2017) reported that adding β‐glucanase and phytase increased the abundance of ileal Lactobacillus abundance while reducing *E. coli* populations in broilers. In the present study, exogenous enzyme supplementation did not significantly affect ileal bacterial counts, possibly due to low enzyme dosage or differences in feed ingredients and formulations used in other studies.

## 5 Conclusion

Conditioning corn at 55°C for incorporation into broiler mash diets represents a novel approach to enhance calcium digestibility and reduce the FCR during the finisher period. Furthermore, the addition of exogenous enzymes to diets containing corn conditioned at 70°C resulted in a significant decrease in FCR. The thermal processing of ground corn at 85°C improved the AME value of the corn; however, neither heat treatment nor enzyme supplementation had a significant impact on the AMEn value. Heat processing of corn also led to an increase in the height of intestinal villi, suggesting improved gut health. Additionally, conditioning corn at 55°C was associated with a reduction in ileal *Clostridium* counts, indicating potential benefits for gut microbiota balance. Overall, these findings highlight the potential of thermal processing and enzyme supplementation to optimise broiler nutrition and performance.

## Author Contributions

**Mohsen Teymouri**: data curation, formal analysis. **Ahmad Hassanabadi**: project administration, supervision, software; review. **Hosna Hajati**: writing and editing. **Abolghasem Golian**: methodology.

## Ethics Statement

The authors confirm that the ethical policies of the journal, as noted on the journal's author guidelines page, have been adhered to and the appropriate ethical review committee approval has been received. The authors confirm that they have followed EU standards for the protection of animals used for scientific purposes.

As part of this experiment, all animal procedures and ethics considerations were performed following the Guide to the Care and Use of Agricultural Animals in Research and Teaching (FASS, 2010). Moreover, this study was conducted according to the procedures established by the Iranian Ministry of Agriculture (Experimental Authorization No. ASRI‐2016‐95014).

## Conflicts of Interest

The authors declare no conflicts of interest.

### Peer Review

The peer review history for this article is available at https://www.webofscience.com/api/gateway/wos/peer‐review/10.1002/vms3.70320.

## Data Availability

It will be available on request.
